# Mechanisms and Mediators of Inflammation: Potential Models for Skin Rejection and Targeted Therapy in Vascularized Composite Allotransplantation

**DOI:** 10.1155/2012/757310

**Published:** 2012-09-19

**Authors:** Theresa Hautz, Dolores Wolfram, Johanna Grahammer, Ravi Starzl, Christoph Krapf, Johann Pratschke, W. P. Andrew Lee, Gerald Brandacher, Stefan Schneeberger

**Affiliations:** ^1^Center for Operative Medicine, Department of Visceral, Transplant and Thoracic Surgery, Innsbruck Medical University, Anichstr. 35, A-6020 Innsbruck, Austria; ^2^Department of Plastic and Reconstructive Surgery, Innsbruck Medical University, Anichstr. 35, A-6020 Innsbruck, Austria; ^3^Language Technologies Institute, Carnegie Mellon University, Pittsburgh, PA 15213, USA; ^4^Department of Plastic and Reconstructive Surgery, Johns Hopkins University School of Medicine, Baltimore, MD 21287, USA; ^5^Department of Cardiac Surgery, Innsbruck Medical University, Anichstr. 35, A-6020 Innsbruck, Austria

## Abstract

Vascularized composite allotransplantation (VCA) is an effective treatment option for patients suffering from limb loss or severe disfigurement. However, postoperative courses of VCA recipients have been complicated by skin rejection, and long-term immunosuppression remains a necessity for allograft survival. To widen the scope of this quality-of-life improving procedure minimization of immunosuppression in order to limit risks and side effects is needed. In some aspects, the molecular mechanisms and dynamics of skin allograft rejection seem similar to inflammatory skin conditions. T cells are key players in skin rejection and are recruited to the skin via activation of adhesion molecules, cytokines, and chemokines. Blocking these molecules has not only shown success in the treatment of inflammatory dermatoses, but also prolonged graft survival in various models of solid organ transplantation. In addition to T cell recruitment, ectopic lymphoid structures within the allograft associated with chronic rejection in solid organ transplantation might contribute to the strong alloimmune response towards the skin. Selectively targeting the molecules involved offers exciting novel therapeutic options in the prevention and treatment of skin rejection after VCA.

## 1. Introduction

Acute skin rejection is a frequent challenge, and long-term immunosuppression is a necessity in vascularized composite allotransplantation (VCA) [1]. The toxicity profile of such a drug treatment includes metabolic side effects, opportunistic infections, malignancy, and organ damage [2–6]. This illustrates the need for immunosuppressive-sparing protocols in order to limit side effects of this quality-of-life improving procedure and widen the indications for VCA. 

The infiltration of alloantigen specific T cells into the skin allograft has been identified as a central element of acute skin rejection in VCA [7, 8]. Histologically, the appearance of skin rejection shares many common features with inflammatory skin diseases and may be difficult to distinguish [9, 10], suggesting that underlying immunological mechanisms might be similar in some aspects. In inflammatory skin conditions, T-cell recruitment to the skin is orchestrated by a multitude of adhesion molecules, cytokines, and chemokines [11]. In part, this concept of inflammation and immune activation holds also true for the initiation and progression of allograft rejection in solid organ transplantation (SOT) [12]. A mechanism currently discussed to be involved in the development of chronic allograft rejection is the formation of lymphoid neogenesis and tertiary lymphoid organs (TLOs) in the transplant [13–15].

The mechanisms and dynamics of skin allograft rejection have been partially understood and remain the subject of numerous trials aiming at a better understanding of the pathophysiology and novel and targeted drug development. We herein review the molecular events and key players of inflammation as well as new therapies with particular regard to skin inflammation and allograft rejection in SOT and discuss them in the light of acute and chronic skin allograft rejection of VCAs.

## 2. Adhesion Molecules: Anchors for Lymphocyte Recruitment to the Skin

Adhesion molecules play a crucial role in the function of immune cells. They are the central actors helping leukocytes to immediately convert from an inactive, nonsticky status to an adhesive status, though permitting adhesion to the vascular endothelium with transmigration to inflamed tissues. Further they support cell-cell interactions through various homophilic and heterophilic interactions and have the ability to transmit costimulatory signals to the interacting cells. The expression pattern of adhesion molecules is characteristic for each cell population and changes during the maturation process of a cell [16].

### 2.1. Adhesion Molecule Families

**(1) Selectins**

3 subtypes of selectins, characterized through their N-terminal lectin domain, are defined [17, 18]: E-selectin is mainly expressed by activated endothelial cells, whereas endothelium of noninflamed tissue does not express E-selectin. Potent stimuli of E-selectin expression are IL-1 and TNF [19]. The “P” in P-selectin stands for “platelet”, but P-selectin is also expressed in activated endothelial cells, where it is stored in Weibel-Palade bodies [20] and is released upon stimulation [21]. In contrast to E- and P-selectins, L-selectin is constitutively expressed on lymphocytes, neutrophils, and monocytes and is known to play a crucial role in homing of lymphocytes to secondary lymphoid tissues through binding to its counter-receptor addressin, which is expressed by high-endothelial venule cells [22, 23]. However, there is now growing evidence that all three types of selectins contribute to leukocyte extravasation in the skin with overlapping effect. E- and P-selectin seem to play the most important role in leukocyte homing into the skin [24]. This idea is supported by the failure of monoselectin antagonists and the success of pan-selectin agonists in targeting leukocyte extravasation [25, 26]. All types of selectins bind to carbohydrate ligands such as the tetrasaccharides Sialyl-Lewis-x or P-selectin glycoprotein ligand-1 (PSGL-1) [27, 28].

**(2) Integrins and the Ig Family**

Leukocytes (neutrophils, monocytes, lymphocytes, and natural killer cells) express the integrins lymphocyte function-associated antigen-1 (LFA-1) and Mac-1 (both sharing a common *β*2-subunit [29]), which bind intercellular adhesion molecule-1 (ICAM-1) and ICAM-2, two members of the Ig superfamily expressed by vascular endothelial cells, and leukocytes. While ICAM-1 expression on vascular endothelium and leukocytes can be stimulated [30], ICAM-2 is constitutively expressed on the endothelium as a target for beta2 integrins [31]. Another integrin expressed on mainly lymphocytes and monocytes is very late activation antigen (VLA), which binds to vascular cell adhesion molecule-1 (VCAM-1) on endothelial cells [32]. VCAM-1 has been shown to mediate several steps in the process of leukocyte extravasation. It is not only involved in firm adhesion, but also in rolling of T cells and transmigration through the endothelium [33].

### 2.2. Functions during Inflammation

**(1) Cellular-Vascular Interactions**

an important function of adhesion molecules is the mediation of cellular-vascular interactions, enabling leukocytes in a multistep cascade to exit the blood vessel and to migrate into the inflamed tissue [34]. The selectin-sialyl-Lewis-x interaction between vascular endothelium and the leukocyte first leads to cell rolling through reversible tethers between the leukocyte and the vessel wall. The leukocyte is slowed down and thus brought into closer proximity to the endothelium. A more firm adhesion is required before the effective transmigration can occur: this arrest of the rolling leukocyte is provided through interactions between immunoglobulins and integrins. For the induction of tight adherence, costimulating ligands and chemokines enhance the avidity of integrins on leukocytes [35–37]. For transmigration junctional adhesion molecules (JAMs), which are constitutively expressed at the borders between endothelial cells, play an important role through interaction with VLA-4 or Mac-1 on the leukocyte [38].

**(2) Cell-Cell Interactions**

In addition to cellular-vascular interactions interaction between different types of immune cells and leukocytes is required for an orchestrated cellular immune response. “The immunological synapse” is an assembly of adhesion molecules, which provides relatively stable interactions between cells of the immune system thereby supporting antigen recognition and enabling bilateral stimulation of the cells. One important mode of cell-cell interaction is the binding of naïve T lymphocytes to antigen-presenting cells (APCs) such as dendritic cells (DCs) [39, 40]. The interaction between cells through adhesion molecules allows the T-cell receptor (TCR) to scan the surface of a DC for appropriate major histocompatibility complex (MHC-) displayed peptides [41–43]. Furthermore, the interaction between T-cells and B-cells [44, 45] and T-cell-mediated killing (through T cell-target cell adhesion and as well the natural killer cell-target cell interaction) relies on adhesion molecules [46]. In this context adhesion molecules are not only anchors for the cells, but also transmitters of important costimulatory signals for many immunity-related functions.

### 2.3. Adhesion Molecules in Inflammatory Skin Diseases

In inflammatory skin diseases such as psoriasis, allergic contact dermatitis, and atopic dermatitis, it has been shown that T cells play a central role in the initiation and/or perpetuation of cutaneous inflammation [47, 48]. While the distinct entities seem to be related to environmental as well as genetic factors, they can be uniformly characterized through a subset of T lymphocytes found in inflammatory skin lesions staining positive for cutaneous lymphocyte-associated antigen (CLA) [49]. CLA is a modified carbohydrate ligand interacting with E-selectin during skin homing of these lymphocytes. However, further characterization of this distinct cell population revealed that they are a heterogenous population of CD4+ and CD8+ T cells. It has been shown that CLA-bearing T cells preferentially extravasate through the endothelium of the superficial dermal plexus [50]. Skin homing of T lymphocytes therefore seems to be a central mechanism in inflammatory skin diseases.

For psoriasis the crucial role of T cells homing to the skin has been clearly demonstrated in several in vitro and animal studies [51, 52] and this concept is further supported by the effectiveness of therapies targeting either the number/proliferation or the extravasation of T lymphocytes [53, 54]. The histological pattern of psoriasis shows a hyperproliferation and hyperkeratosis of epidermal keratinocytes as well as cellular infiltration into dermis and epidermis. Epidermal keratinocytes in psoriatic lesions have been shown to display upregulation of MHC class II antigens as well as induced expression of ICAM-1. Furthermore, the vascular endothelial cells upregulate adhesion molecules of all classes: E-selectin, ICAM-1, VCAM-1, and MHC class II antigens [55]. The understanding of the relevance of lymphocyte homing into the skin for the development of psoriasis has initiated a quest for potential treatments [56]. However, the redundancy and the many overlapping functions of adhesion molecules have made the development of effective therapeutics difficult [57]. The insufficient therapeutic potency of several substances such as monoclonal antibodies against E-selectin has revealed the difficulty of this therapeutic approach.

Nevertheless, there is a strong need for new anti-inflammatory substances for the treatment of inflammatory skin diseases and induction of long-lasting remissions. The integrin inhibitor efalizumab (Raptiva), which is a monoclonal antibody against the alpha-subunit of LFA-1, has clinically shown to alleviate skin inflammation in plaque psoriasis [58]. However, EMEA and FDA recommended withdrawal of this substance from the market because of a severe side effect: progressive multifocal leukoencephalopathy was observed in a few patients. Numerous compounds have been introduced inhibiting selectin function. Efomycine M [59], BMS-190394 [60], OJ-R9188 [61], and TCB-1269 [62] showed an effect in preclinical models of psoriasis, delayed-type hypersensitivity (DTH), and atopic dermatitis. However, only insufficient response was reported for most inhibitors in phase I/II trials. Other substances, which are still being evaluated in preclinical trials, include inhibitors of fucosyltransferase IV [63] (an enzyme which modifies carbohydrate ligands to CLA, a high-affinity ligand for E-selectin). Furthermore, alefacept, a fusion protein of LFA-3 and the Fc-portion of human IgG, has been reported to cause long-term remission in at least a subpopulation of psoriasis patients [64].

### 2.4. Adhesion Molecules in Solid Organ Transplantation

Ischemia-reperfusion injury (IRI) is an event of excessive inflammatory response that occurs after temporary absence of blood supply, such as shock, infarction, and transplantation. Key events during IRI are the generation of damage-associated molecular patterns (DAMPSs) and upregulation of inflammatory cytokines and adhesion molecules, which contributes to recruitment of leukocytes [65, 66]. In liver transplantation IRI affects the outcome and results in 2–10% early graft failures [67]. Moreover, it has been speculated that IRI may also lead to higher incidences of acute and chronic rejection. Gene expression profiling of IRI in human liver allografts has revealed an upregulation of adhesion molecules and integrins [68]. Several preclinical and clinical trials have focused on prevention of IRI in SOT, and blocking of adhesion molecules has shown promising results in many models. In the setting of liver transplantation, blocking P-selectin with a monoclonal antibody resulted in decreased incidence of IRI in mouse models [69, 70] and most recently in a clinical phase II study [71]. The leukocyte adhesion cascade in myocardial IRI remains an interesting target for therapeutic intervention. Molecules such as B*β*15–42 [72] and FX06 [73] have shown promise for limiting damage in myocardial IRI.

As the mechanisms of leukocyte recruitment to the allograft in the course of rejection is similar to leukocyte recruitment during inflammation [12, 74], strategies to block adhesion molecules also demonstrated effects on allograft survival in different settings. inhibition of LFA-1 prolonged graft survival in murine heart allotransplantation [75, 76]. Prolonged allograft survival was achieved in an islet transplant model in nonhuman primates [77]. The monoclonal antibody against LFA-1, efalizumab, has demonstrated efficacy in a clinical phase I/II study of renal transplantation [78]. However, an increased incidence of posttransplant lymphoproliferative disorder was observed in these patients [79]. A study published by Langer et al. [80] in 2004 showed prolonged survival of rat kidney allografts using the selectin inhibitor OJ-R9188. This effect was mainly due to a reduction of infiltrating T cells and macrophages as well as decreased intragraft expression of cytokines and chemokines.

### 2.5. Adhesion Molecules in Vascularized Composite Allotransplantation-Potential Targets for Therapy

We have recently published an analysis of more than 170 biopsies taken from five human hand and forearm transplant recipients demonstrating the upregulation of adhesion molecules during skin rejection [7]. Immunohistochemical staining of skin samples has revealed a strong correlation of LFA-1 (also found to be expressed in keratinocytes), ICAM-1, and E-selectin with the severity of rejection, while none of these markers was found to be upregulated in nonrejecting skin. Quantitative PCR analysis, however, showed no correlation between the severity of rejection and the gene expression of these molecules, which may indicate that these adhesion molecules are not solely regulated at the gene level.

In a series of experimental studies using a rat hind-limb transplant model, adhesion molecule blockers were administered subcutaneously (SC) into the allograft after a short course of systemic immunosuppression (tacrolimus) to prevent rejection. Targeting E- and P-selectins using the small-molecule inhibitor Efomycine M resulted in long-term (150 days) allograft survival in 5 out of 6 animals [7]. Histology on day 150 showed a mild lymphocytic infiltrate in the dermis and only single vacuolized keratinocytes in the epidermis. Local intragraft administration of anti-ICAM-1 and anti-LFA-1 significantly prolonged graft survival when compared to controls. In 3 out of 4 animals long-term graft survival was achieved (paper in preparation). In another attempt to address local inhibition of adhesion molecules, the fibrin derivative B*β*15–42, which blocks VE-cadherin, revealed a statistically significant prolongation of hind-limb allograft survival in the rat when combined with subtherapeutic doses of tacrolimus. When local treatment with B*β*15–42 was then combined with an induction with IL-2 Fc and a short course of cyclosporin A, long-term allograft survival with significant reduction of CD4+ and CD8+ T cells was achieved (paper in preparation). These data indicate the potential of leukocyte migration blockers to prevent skin rejection in a rat VCA model.

## 3. Cytokines and Chemokines as Important Mediators for Cell Trafficking 

Attraction of mononuclear cells to sites of inflammation does not only require membrane-bound adhesion molecules but also a close interplay of the inflammatory signal presented by a variety of soluble or membrane-borne chemoattractive factors. It is known that the specific expression pattern of chemokines and their receptors determines the type of cell that is attracted to the inflamed tissue. This pattern of chemokines is regulated by the local cytokine milieu. For example, interferon-*γ* (IFN-*γ*) induces upregulation of chemokines, which subsequently attracts neutrophils, monocytes and T helper-1 (Th1) cells. Further, a T helper-2 (Th2-) dominated cell recruitment pattern is induced by chemokines upregulated upon exposure to IL-4 and IL-13 [81].

Chemokines can be characterized as a family of cytokines with chemotactic activity for leukocytes. To this day, approximately 60 chemokine members have been identified. They are divided into C, CC, CXC, CX3C subfamilies based on the cysteine motifs near the aminoterminal end of the molecule [82]. Several studies emphasize the importance of chemokines and their receptors in the allograft rejection process and their role in leukocyte recruitment, Th1 and Th2 cell differentiation and DC movement and maturation [83–87]. Studies on human renal biopsies delineated that the expression of Th1 chemokine receptors (CCR5 and CXCR3) and their ligands (CXCL10 (=IP10), CXCL9 (=Mig) and CCL5 (=RANTES) is associated with acute rejection [88]. Mig was increased in a lung transplant model and its inhibition decreased intragraft migration of mononuclear cells [89]. The importance of CCR5 was shown in islet allografts since targeting CCR5 resulted in significant prolongation of these grafts [90]. Increased CXCR3 expression was demonstrated in a murine skin allograft model during rejection and peptide nucleic acid (PNA) CXCR3 antisense significantly prolonged allograft survival by blockade of CXCR3+ T-cell infiltration into the allograft [91]. Li et al. [92] investigated the intragraft expression profile of 11 chemokines from all four chemokine subfamilies in a murine skin transplantation model and demonstrated that CCL5/RANTES, CCL17/TARC, and FKN were expressed at equivalent levels in iso- and allografts. The expression of eight chemokines was upregulated in allografts compared with isografts also in dependence of postoperative days. The most significantly elevated chemokine was I-TAC (CXCL11), which peaked during rejection (postoperative day 7), and when inhibited via intradermal injection of anti-I-TAC monoclonal antibody significantly prolonged skin allograft survival. Most studies in transplantation have concentrated on rather few chemokines. To analyze their roles in a meaningful manner, novel techniques including commercially available multiprobe ribonuclease protection assays, antichemokine and antichemokine-receptor monoclonal antibodies, and gene-knockout animals are now available [93, 94]. Despite these prospects, it is important to emphasize that many data from in vitro experiments demonstrated the presence of multiple ligands for one chemokine receptor and often multiple receptors for one chemokine. This may help to explain, why allograft rejection was not abrogated in any of these trials. Thus a cocktail of reagents directed to multiple recruiting chemokines may be required for efficient inhibition of T-cell infiltration into allograft. In this context we believe that this may also provide a future promising strategy in VCA.

## 4. Mediators of Inflammatory Skin Diseases: Parallels to Skin Rejection

Skin rejection in VCA presents with erythematous macules that may progress if not treated to scaly violaceous lichenoid papules covering the complete surface of the graft [8]. These alterations are not specific for rejection and may mimic inflammatory dermatoses. Kanitakis [10] emphasized the diagnostic challenges in early or mild skin rejection; differentiation from contact dermatitis, insect bites or dermatophyte infections may be difficult in these stages.

Parallels between acute skin rejection and inflammatory dermatoses (e.g., contact dermatitis, psoriasis, and atopic dermatitis) also exist on the molecular and Cellular level. Allergic contact dermatitis for example is a T-cell-mediated DTH reaction that occurs upon hapten stimulation in sensitized individuals [95]. Therefore, the differentiation by histological and macroscopic criteria can be difficult. It has been demonstrated, that T cells (CD4+ and CD8+ cells) are critical and that elements of the innate immune system (e.g., natural killer cells) may play a key role [96]. Epidermal Langerhans cells as the most competent APCs in the skin as well as keratinocytes are regulating this inflammatory process. Cytokines derived from Langerhans cells (e.g., IL-12) and from T cells (IFN-*γ*, IL-4, and IL-10) play a pivotal role in the induction and initiation of this skin disease [97].

Given the close interaction of chemokines in the inflammatory process and immune response, it is not surprising that a number of dermatological diseases are a result of chemokine dysregulation [98]. Strong chemokine expression in allergic and inflammatory skin diseases such as psoriasis and contact hypersensitivity (CHS) has been documented [99–102]. Specifically, CXCL8/IL-8 and the related CXCL2/Gro-*β* are significantly upregulated in psoriatic skin lesions and thus responsible for the typical intraepidermal aggregation of neutrophils. CCL2/MCP-1 and CCL5 are responsible for attracting predominately monocytes and T cell subsets, and CXCR3 ligands attract Th1 cells [103]. The expression of cytokines and chemokines during the sensitization and elicitation phase of CHS has been well studied [104]. Watanabe et al. [105] has shown that TNF-*α* and IL-1*β* play a main role in the sensitization phase of CHS, meanwhile the elicitation phase is predominately characterized by IFN-*γ*, IL-1, IL-4, and TNF-*α* expression.

## 5. Tertiary Lymphoid Organs: Do They Play a Pivotal Role in Chronic Rejection of VCAs?

The role of chronic rejection in VCAs is poorly understood so far. As reconstructive transplantation is a relatively young field, follow-up periods of VCA recipients are currently limited to 13 years. Allograft vasculopathy is the main feature in chronic rejection of solid organ allografts. Only a limited number of reports on vascular changes of graft vessels in a VCA are available at this time [106, 107]. It is hypothesized that multiple (untreated) acute rejection episodes imitate a state of chronic inflammation, which may trigger myointimal proliferation and occlusion of allograft vessels [106, 108].

TLOs are lymphoid-like structures that can be found in chronically inflamed tissues [109]. They are composed of B- and T-cell aggregates, specialized populations of DCs, well-differentiated stromal cells, and high endothelial venules (HEVs), but they are not encapsulated [110]. Many of the molecular signals and events leading to the development of secondary lymphoid organs have been shown to be as well involved in the formation of TLOs [14, 111]. Mesenchymal lymphoid tissue organizers express CXCL13, MAdCAM, ICAM, and VCAM and thereby recruit CD4+CD3− haematopoietic lymphoid tissue inducers. The expression of lymphotoxin on these inducer cells further upregulates chemoattractants and adhesion molecules via a positive feedback loop, resulting in recruitment of immune cells and formation of HEVs.

### 5.1. TLOs in Chronic Allograft Rejection

The formation of ectopic lymphoid structures is thought to enhance the efficiency of alloantigen presentation and generation of alloreactive lymphocytes and might therefore enhance the alloimmune response. This is speculated to be a mechanism in several chronic inflammatory conditions, such as rheumatoid arthritis, Sjögren's syndrome, and Hashimoto's thyroiditis [112–114]. A retrospective analysis of 350 renal allografts revealed the formation of regional inflammatory infiltrates consisting of T and B lymphocytes, plasmocytoid cells, and DCs [115]. The authors found a strong correlation between the formation of TLOs and an increased incidence of chronic rejection and graft loss. Baddoura et al. [13] reported lymphoid neogenesis in murine cardiac allografts in the course of chronic rejection. 78% of chronically rejected allografts revealed either classical TLOs with organized T- and B-cell zones and peripheral node addressin+ (PNAd+) HEVs or PNAd+ HEVs without organized lymphoid accumulations. Interestingly, the architecture of TLOs has been shown to be related to the immune activation status of the host [116]. In an attempt to address the role and function of TLOs during rejection Nasr et al. [117] reported that TLOs are able to generate effector and memory T cells. In a murine transplantation model full thickness skin grafts containing TLOs due to transgenic expression of lymphotoxin-a (RIP-LTa) were transplanted to recipients lacking all secondary lymphoid organs. These allografts were rejected, while wild-type allografts were accepted. When RIP-LTa and wild-type allografts were transplanted simultaneously both were rejected. Furthermore, Thaunat et al. [118] demonstrated the production of alloantibodies specific for donor MHC class I molecules in germinal centers of TLOs in a rat aortic interposition model, suggesting a local antibody-mediated alloimmune response. In a mouse model of autoantibody-mediated cardiac allograft vasculopathy administration of a lymphotoxin blocker, LT*β*R-Ig fusion protein, abolished allograft TLO formation and inhibited the effector humoral response [119]. Taken together these findings suggest that TLOs in allografts are not only a result of the chronic inflammatory stimulus, but also a site where the alloimmune response is being executed and enhanced.

This contrasts findings by Brown et al. [120], who reported the presence of TLOs in a murine kidney allograft model of tolerance to be associated with superior graft function and survival. In summary, it remains unclear at this point whether TLOs are associated with a destructive or beneficial response in organ and tissue transplantation and if they should be targeted or induced in order to promote long-term graft survival.

## 6. Conclusion

A perivascular infiltrate of mainly CD3+ T lymphocytes in the dermis marks the advent of skin rejection in VCA [9, 121]. The cellular infiltrate then further spreads into the dermis and epidermis leading to dermal-epidermal separation and necrosis if not treated successfully. Since a variety of adhesion molecules as well as cytokines and chemokines are responsible for lymphocyte trafficking towards the epidermis during acute rejection, selectively blocking leukocyte recruitment to the site of inflammation seems a promising approach to prevent and also treat skin rejection in VCA. Moreover, novel concepts targeting intragraft lymphoid neogenesis and the formation of TLOs might be considered in the treatment of chronic allograft rejection, while it remains a puzzling feature in VCA. Targeted therapy, inspired by the novel treatments for inflammatory skin diseases, could evolve to a promising treatment option for VCA patients, lowering their burden of long-term systemic immunosuppression.
